# Numerical simulation of the dynamic distribution characteristics of the stress, strain and energy of coal mass under impact loads

**DOI:** 10.1038/s41598-020-74063-3

**Published:** 2020-10-08

**Authors:** Hongqing Zhu, Shuhao Fang, Yilong Zhang, Yan Wu, Jinlin Guo, Feng Li

**Affiliations:** 1 School of Emergency Management and Safety Engineering, China University of Mining and Technology-Beijing, Beijing, 100083 China; 2 State Key Laboratory Coal Resources and Safe Mining, China University of Mining and Technology-Beijing, Beijing, 100083 China

**Keywords:** Mineralogy, Petrology, Coal

## Abstract

To research the dynamic response characteristics of coal mass under impact loads, based on LS-DYNA software, rigid body bars are simulated to impact coal mass under different speed conditions, and the dynamic distribution characteristics of the stress, strain and energy of coal mass are analyzed. The results demonstrate that (1) the peaks of the axial and radial stresses and strain on the central axis and the radial line obey the power function distribution; at the same position, the axial and the radial stress peaks are close, and the axial strain peak is from much larger than the radial strain peak to close to. (2) The axial and radial stresses generate tensile stresses in the axial and radial propagation directions, respectively, and the coal mass is prone to damage under tensile stress. (3) When the speed is large, the axial stress–strain curve is similar to that of the dynamic load experiment. The axial stress peak, axial strain peak, critical effective stress, critical time and secant modulus have a linear relationship with the velocity. (4) When the dynamic load is large, most of the energy is in the form of kinetic energy, and the total energy loss also increases.

## Introduction

Coal has an important position in energy consumption^1,2^. The dynamic disasters of coal mines such as coal and gas outbursts and rock bursts seriously threaten the safe operation of coal mines^3–7^, and the dynamic disasters of deep coal seams are severe^8–10^. Dynamic load response characteristics are substantial for safe mining^11^. Understanding the damage law of coal mass is of great importance for ensuring the safety of coal mines^12,13^.

Scholars have done a large number of dynamic and static experiments on coal to explore the mechanism of coal and rock dynamic disasters such as coal and gas outbursts and rock bursts. The damage mechanism of coal mass under static loads is relatively established^14–23^, mainly focusing on damage due to shear and compression^24–26^. The damage mechanism of a coal mass under dynamic loading has been modeled with a split-Hopkinson pressure bar (SHPB)^27–32^. The SHPB experiment on coal at impact speeds of 4.174 ~ 17.652 m/s shows that the stress state of coal has a greater impact on electromagnetic radiation than strain and destruction^33^. Based on the SHPB system, Feng et al.^34^ analyzed the mechanism of energy dissipation of coal under dynamic loads. Wang et al.^35^ analyzed the effect of water on the fracture surface of coal using the SHPB experiment. Yin et al.^36^ obtained that the dynamic compressive strength of gas-containing coal under coupled load decreases with increasing initial gas pressure using the SHPB experiment of coal samples. Kong et al.^37^ used the SHPB experiment of coal samples to determine that the dynamic strength and failure strain increased with increasing confining pressure. Ai et al.^38^ concluded that the fractal dimension of cracks on the coal surface increased in the fracture process under SHPB loading. Yang et al.^39^ used the SHPB experiment of coal samples to obtain a linear relationship between the dynamic compressive strength and the applied strain rate. Li et al.^40^ theoretically analyzed the propagation process of impact stress waves in structural coal, but did not describe the dynamic process of damaged coal. Tahmasebinia et al.^41^ proposed a new damage model between the rock and coal messes based on the modified thermomechanical continuous constitutive model, which can predict the possibility of coal burst. Viljoen et al.^42^ analyzed the impact of the internal structure of a coal sample on coal breakage based on impact experiments with coal particles.

Numerical simulations can reproduce the entire dynamic process compared to experiments, and it is easy to monitor changes at various points in a numerical simulation^43^. Xia et al.^44^ used particle simulations to study the effects of different loading wave forms on rock damage from the mesoscale. The process of dynamic breakage and damage evolution of barre granite was reproduced using the explicit hydrocode, ANSYS/LS-DYNA^45^. Wang, Zhang and Zuo et al.^46–48^ simulated the cracks generated by the dynamic compression and tensile failure of coal based on LS-DYNA. Zhao, Zhai and Ye et al.^49–51^ applied LS-DYNA software to the analysis of crack propagation caused by coal blasting. Majidi et al.^52,53^ used LS-DYNA software to study the properties of Holmquist-Johnson–Cook (HJC) model concrete. Meng and Liu et al.^54,55^ simulated the erosion effect of HJC model rock under impact loading based on LS-DYNA. Yuan et al.^56^ performed a numerical simulation of the rock SHPB experiment with the HJC model based on LS-DYNA. Xie et al.^57^ studied the parameters of the HJC constitutive model of coal samples based on coal sample experiments.

At present, the dynamic load damage mechanism of coal mass is not very clear. The damage law and crack evolution mechanism of a coal mass under dynamic loading require further study. The SHPB experiment mainly studies the failure stress and final failure morphology of cylindrical coal mass under impact loading. Due to the limitation of experimental conditions, it is difficult to monitor the dynamic process of the parameter changes of all internal points of an experimental sample during the experiment. Numerical simulations have advantages in this respect. Taking the impact of the cylindrical coal sample in the SHPB experiment as a reference, numerical simulation studies the impact of a small rigid rod on a large coal sample. Based on the LS-DYNA software HJC model, the dynamic distribution characteristics of the stress, strain and energy at various points of coal mass under the impact of a rigid bar at different impact speeds are simulated and analyzed. This study has certain reference significance for explaining dynamic disasters such as coal and gas outbursts.

## Modeling and analysis

### HJC constitutive model

The coal mass used the HJC dynamic constitutive model in LS-DYNA^58,59^. The model mainly contains the strength equation, damage evolution equation and state equation. The ANSYS finite element analysis software subroutine LS-DYNA models a crack in the structure simulation and becomes a discontinuous medium. The program generates cracks in the structure through the failure of a unit. The material model with the failure mode defined in the simulation is through *MAT-ADD-EROSION, which adds a unit failure basis. When the stress and strain of the unit in the finite element model exceed the set value, the unit fails, the failed unit is removed from the model, and multiple deleted units penetrate, forming a crack in the structure.

The literature^60,61^ gives the HJC model parameters of concrete, and the literature^62^ explains the meaning of the HJC model parameters. The literature^63^ gives the HJC model parameters of coal, which only simulate the shape of the impact damage of cylindrical coal sample, and compares it with the failure mode of coal damaged due to impact in the experiment. The literature^57,63^ has determined the HJC model parameters of coal through experiments and formulas, as shown in Table 1.

**Table 1:** HJC model parameters of the coal.

Parameters	Value
${\rho }_{0}$: density	1352 $\mathrm{kg}\bullet {\mathrm{m}}^{-3}$
*G*: shear modulus	0.58 GPa
*K*_*1*_: Constant used for the material with no voids	85 GPa
*K*_*2*_: constant used for the material with no voids	− 171 GPa
*K*_*3*_: constant used for the material with no voids	208 GPa
*D*_*1*_: damage constant	0.027
*D*_*2*_: damage constant	1
*A*: the normalized cohesive strength	0.4
*B*: normalized pressure hardening coefficient	0.7
*C*: Strain rate coefficient	0.05
*N*: pressure hardening exponent	0.5
*T*: tensile strength	1.86 MPa
*P*_*min*_: minimum failure pressure	− 0.3 MPa
${\sigma }_{max}$: failure principal stress	10 MPa
*S*_*max*_: normalized maximum strength	7 MPa
*f*_c_*: quasi-static uniaxial compressive strength of coal	9 MPa
*P*_*c*_: pressure	3 MPa
*u*_*c*_: volumetric strain	0.0008

Only changing the density in the parameters of the HJC model of coal was carried out for many numerical simulation tests, and finally the density with better effect was selected as 1570 kg∙m^−3^.

### Modeling

Refer to Table 2 for the rigid body model parameters of the impact bar.

**Table 2:** The rigid body model parameters.

$\mathrm{Density}$	Elastic modulus	Poisson’s ratio
7900 $\mathrm{kg}\bullet {\mathrm{m}}^{-3}$	210 GPa	0.3

The units of the physical quantities used in the LS_DYNA program simulation is shown in Table 3.

**Table 3:** The units of the physical quantities.

Quality	Time	Length	Stress	Density	Velocity	Energy
g	$\mathrm{\mu s}$	cm	exp11 Pa	exp3 kg/m^3^	exp4 m/s	exp5 J

**Figure 1:**
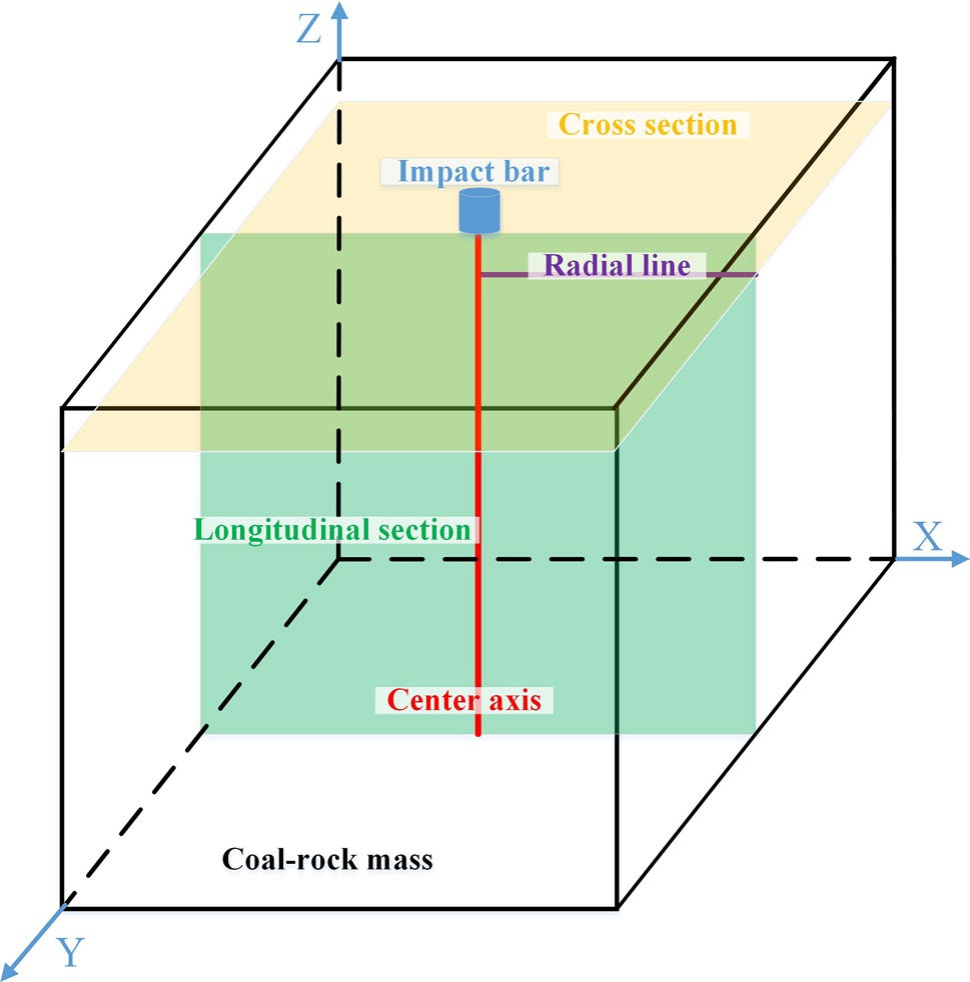
The model schematic diagram.

The model size considers the following conditions: the ratio of the rigid bar to the coal mass is 0.1, the size of the coal sample used in the general experiment is 5 cm, and the computing power of the computer. The model was established with the following: a cubic shape of a coal mass with a side length of 50 cm, and a cylindrical impact bar with a diameter of 5 cm and height of 5 cm. The impact bar was located at the center of the top surface of the coal mass and vertical to the top surface. The longitudinal section passed through the central axis of the coal mass, vertical to the Y-axis, and the cross section was vertical to the Z-axis and 4.5 cm high. The radial line was the intersection of the cross section and the longitudinal section. The radial line, central axis cross section and longitudinal section of the coal mass on the model are shown in Fig. 1.

The coal mass bottom plane set the vertical displacement constraint and the boundary condition without reflection. The model adopted the three-dimensional solid element (Solid164). The impact bar adopted the rigid body material model, and the coal mass was established by the HJC material model. The models were all meshed by the hexagonal mapping method, and the meshing of the coal mass was refined. Refer to the speed in the SHPB experiment, after many simulation tests, and finally select a representative impact bar speed. The dynamic response characteristics of the impact bar impacting the coal mass at speeds of 1 m/s, 4 m/s, 10 m/s, 20 m/s, 30 m/s, 50 m/s, and 100 m/s were simulated.

After the above conditions are set step by step, the different speeds of the impact bar are given for numerical calculation. After the numerical simulation calculation starts, the impact bar will impact the coal mass at the given speed. After the operation is over, the simulation process and related calculation result parameters can be viewed through post-processing.

### Mechanism analysis

The kinetic energy of the impact bar equation can be expressed as:

1 $${E}_{k}=\frac{1}{2}m{v}^{2}=\frac{1}{2}\rho \pi {r}^{2}h{v}^{2}$$

where ${E}_{k}$, *m*, *v*, $\rho$, *r,* and h are defined as the kinetic energy, mass, density, radius and height of the impact bar, respectively. The parameters of the impact bar and the impact velocity are substituted into Eq. (1) to obtain the corresponding kinetic energy of the impact bar.

When the impact bar impacts the coal mass, the stress on the coal mass in contact with the impact bar is concentrated, and plastic deformation occurs. The wave impedance (*ρc*_*l*_) of the plastic coal is smaller than the wave impedance (*ρc*_*e*_) of the elastic coal, and the velocity of the coal mass particle is expressed as:

2 $$v=\frac{-\sigma }{\rho c}$$

Because $\rho \nu {c}_{1}<\rho \nu {c}_{e}$, the impact stress wave is transmitted and reflected at the interface of the elastic and plastic coal bodies, so the stress behind the direction of propagation of the stress wave is smaller than that at the front, where tensile stress is generated and the coal mass is easily damaged.

## Axial and radial stress and strain

**Figure 2:**
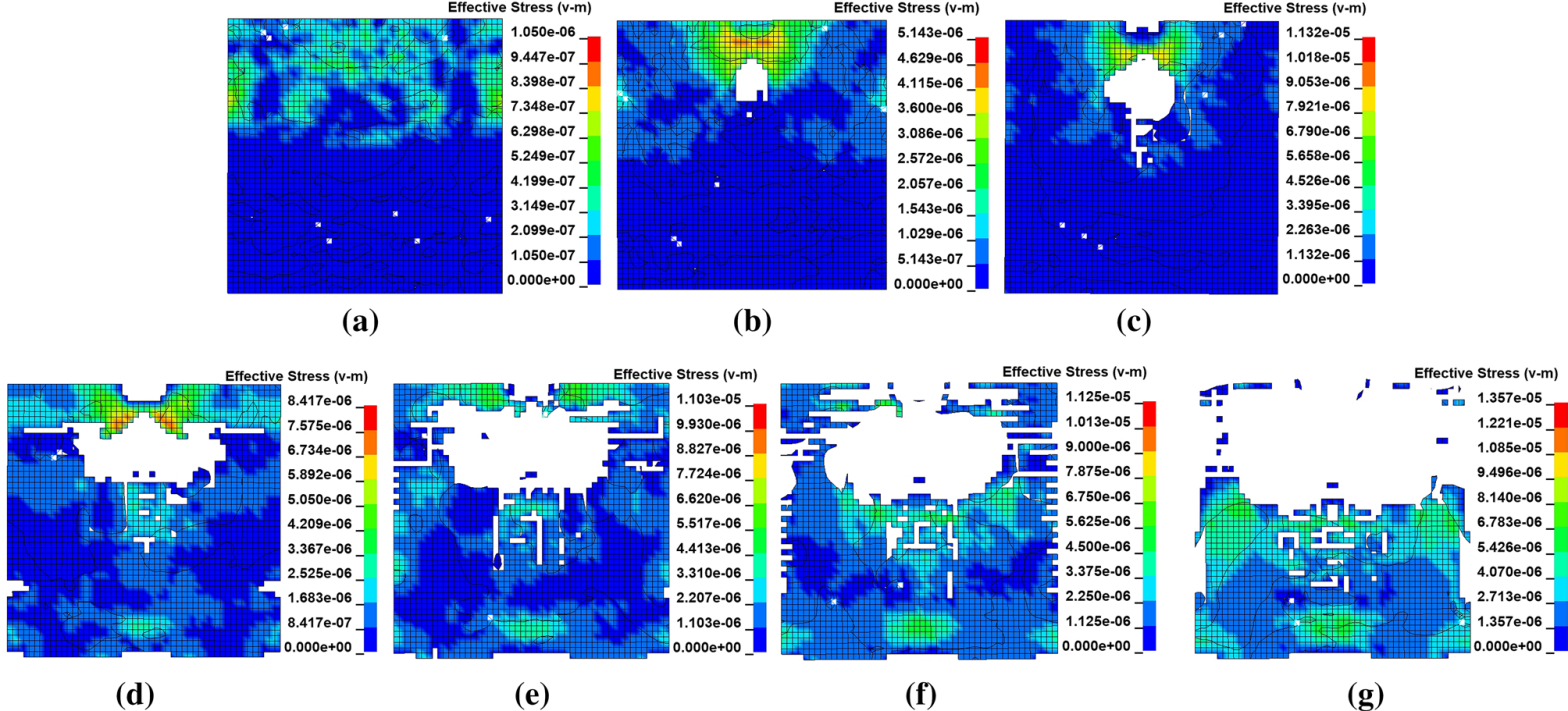
Distribution of the effective stress on the longitudinal section.

The set time for each simulation is 500 μs, and the final effective stress distributions on the longitudinal section at impact bar speeds of 1 m/s, 4 m/s, 10 m/s, 20 m/s, 30 m/s, 50 m/s, and 100 m/s are shown in Fig. 2a–g.

When the velocity is greater than 4 m/s, internal damage to the coal mass occurs; when the velocity is greater than 20 m/s, external damage to the coal mass occurs; when the velocity is 100 m/s, the coal mass is seriously damaged. Only the internal damage of the coal mass is analyzed at a speed of 10 m/s, and the severe damage of the coal mass is analyzed at a speed of 100 m/s.

### Axial and radial stress and strain distributions at 10 m/s

**Figure 3:**
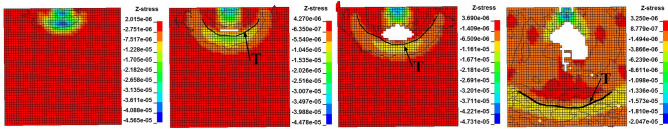
Dynamic distribution of the axial stress on the longitudinal section at a velocity of 10 m/s.

The dynamic distribution of the axial stress on the longitudinal section of the coal mass at a speed of 10 m/s is shown in Fig. 3. The maximum axial stress is located in the center of the contact surface and exhibits spherical downward propagation. The axial stress is compressive stress in the axial direction. When the axial stress at the apex of the coal mass exceeds the peak value, the axial stress behind the direction of propagation of the axial stress is smaller than that at the front; that is shown in the T area of Fig. 3, where the axial stress becomes tensile stress, and the coal mass is prone to damage. This is consistent with the theoretical analysis.

The coal mass is plastically deformed where the stress wave arrives, and the stress wave is reflected at the elastic and plastic interface. Therefore, the stress behind the direction of propagation of the stress wave will be smaller than that at the front, and tensile stress will be generated here.

**Figure 4:**
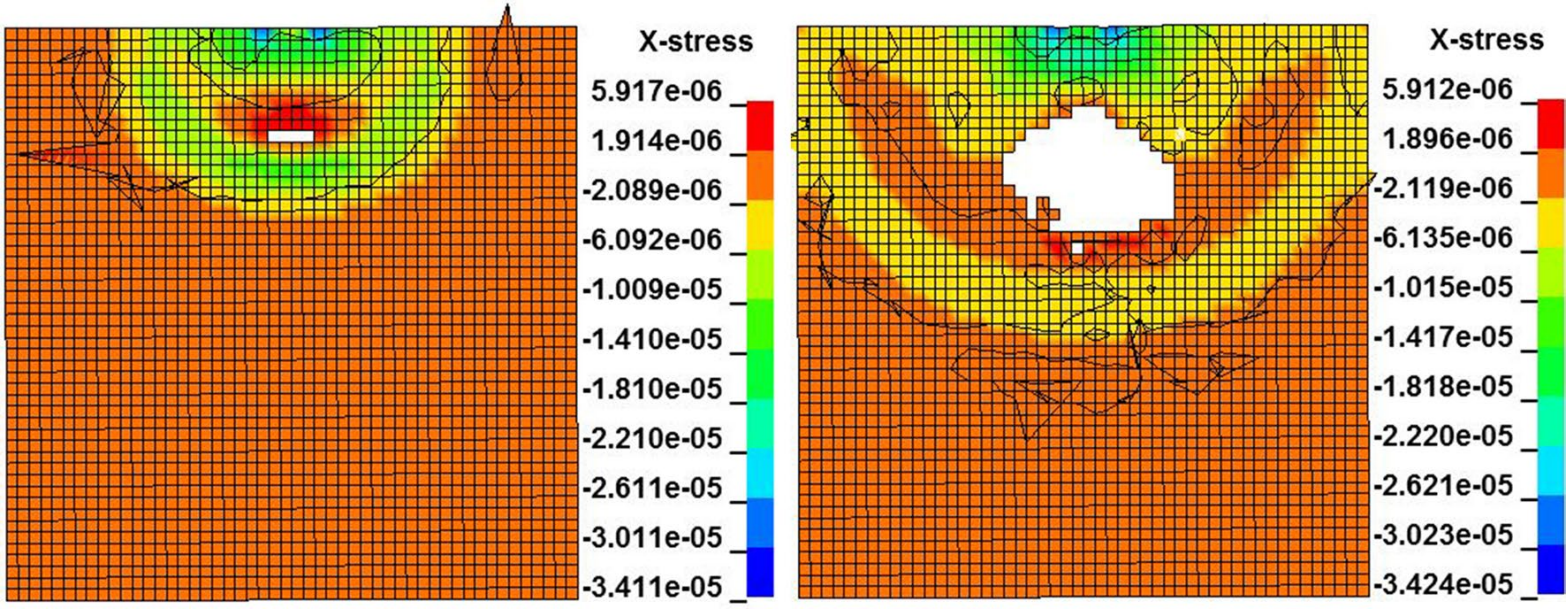
Dynamic distribution of the radial stress on the longitudinal section at a velocity of 10 m/s.

The dynamic distribution of the radial stress on the longitudinal section of the coal mass at a speed of 10 m/s is shown in Fig. 4. The radial stress distribution is similar to the axial stress distribution.

**Figure 5:**
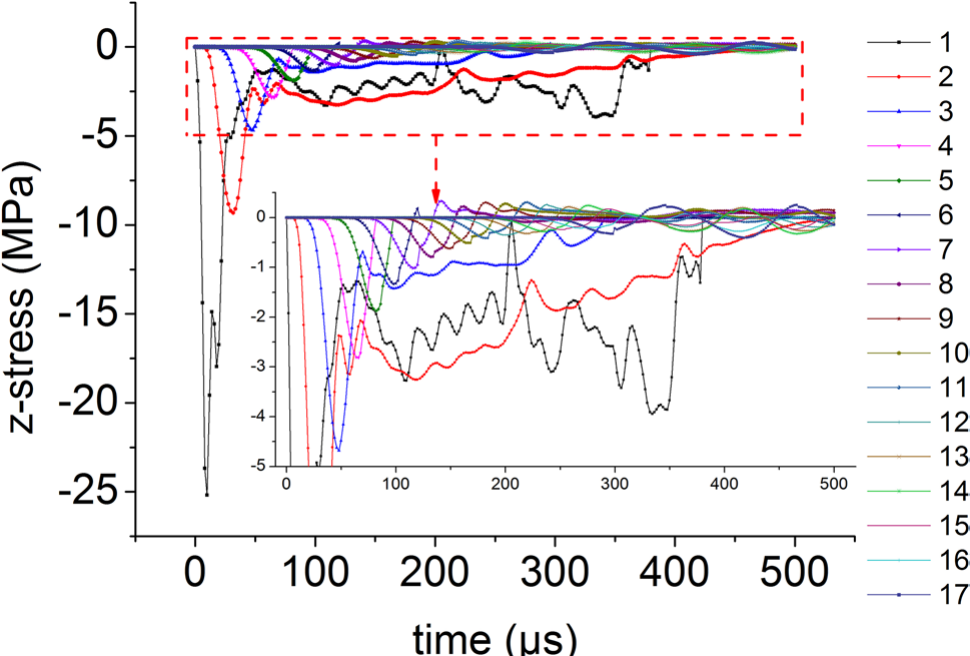
The axial stress versus time curve.

On the central axis of the coal mass, seventeen element points are equidistantly selected from the top to the bottom. The axial stress versus time curve of the central axis element points when the impact bar speed is 10 m/s is shown in Fig. 5. Except for the top three element points, the axial stress peak on the central axis propagates from top to bottom, the axial stress peak decreases continuously and the axial stress behind the axial stress propagation direction is smaller than that at the front.

**Figure 6:**
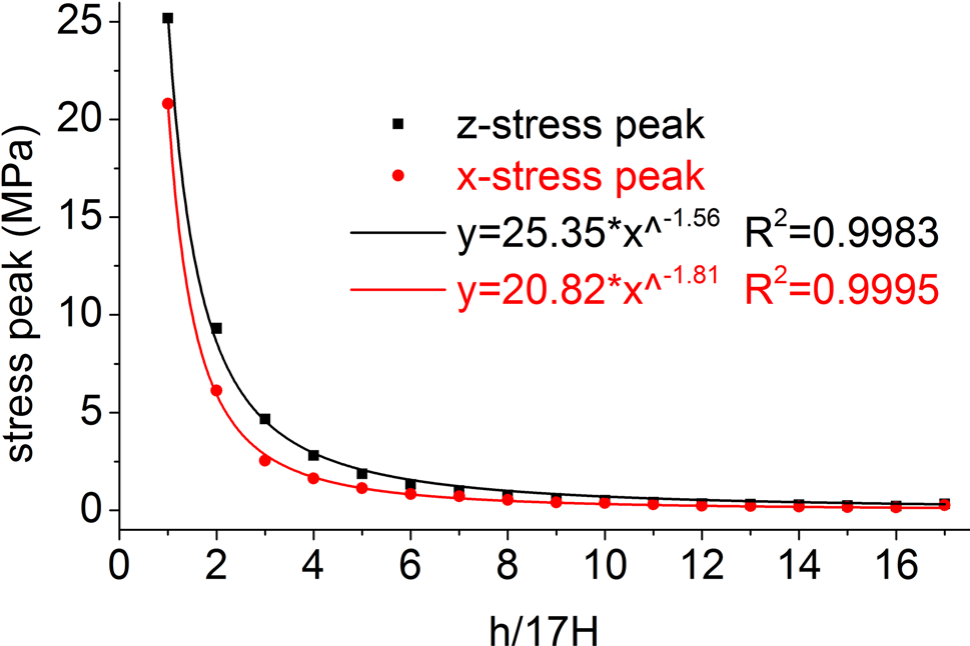
Distribution of the axial and radial stress peaks.

**Figure 7:**
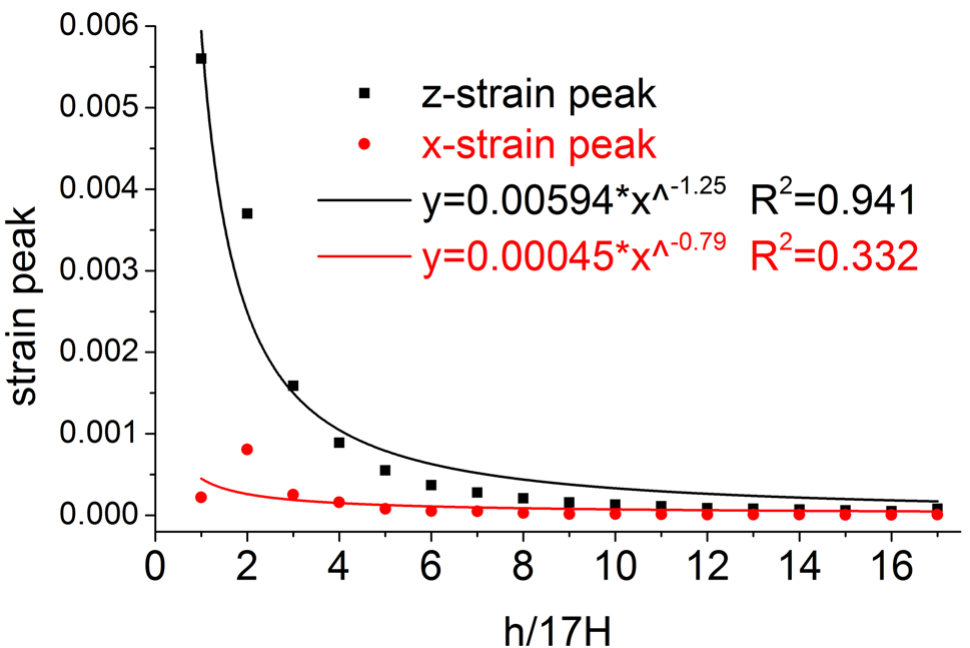
Distribution of the axial and radial strain peaks.

The distribution of the axial stress peak, radial stress peak, axial strain peak, and radial strain peak at seventeen element points on the central axis of the coal mass are shown in Figs. 6 and 7, respectively. The axial stress peak, radial stress peak and axial strain peak distribution of the central axis are in accordance with the power function of the Allometric1 function model, and the correlation coefficient squares (*R*^2^) are 0.9983, 0.9995 and 0.941 respectively. The radial strain distribution does not conform to the power function. At the same position, the axial stress peak and the radial stress peak are close, and the axial strain peak on the central axis is from far greater than the radial strain peak to close to.

**Figure 8:**
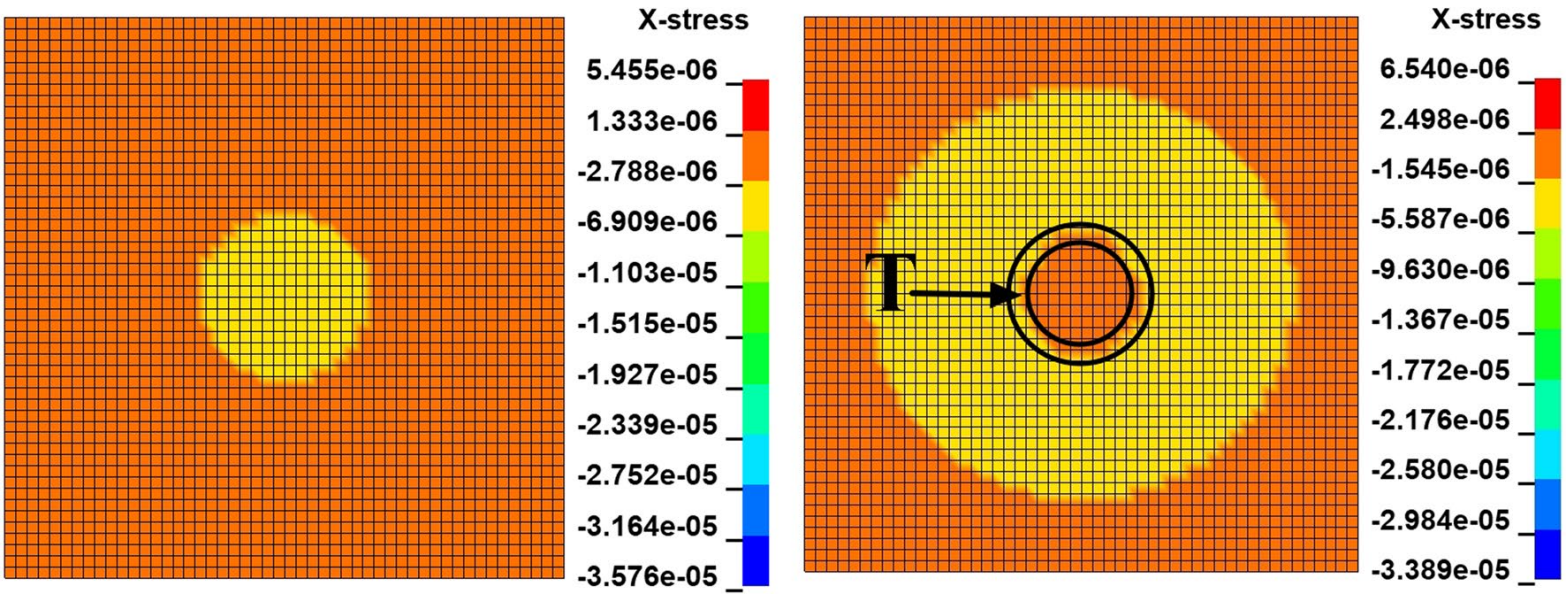
Dynamic distribution of the radial stress in the cross section at a velocity of 10 m/s.

The radial stress distribution on the cross section of the coal mass is shown in Fig. 8. The maximum radial stress is at the center of the circle. The radial stress is compressive stress in the radial direction. When the radial stress at the center point exceeds the peak value, the radial stress behind the direction of propagation of the radial stress is smaller than that at the front; this is shown in the T area of Fig. 8, where the radial stress becomes tensile stress, and the coal mass is prone to damage. This is consistent with the theoretical analysis.

**Figure 9:**
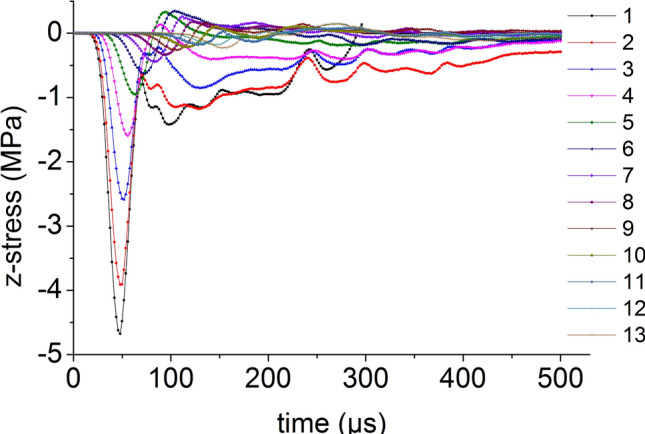
Axial stress and time relationship.

**Figure 10:**
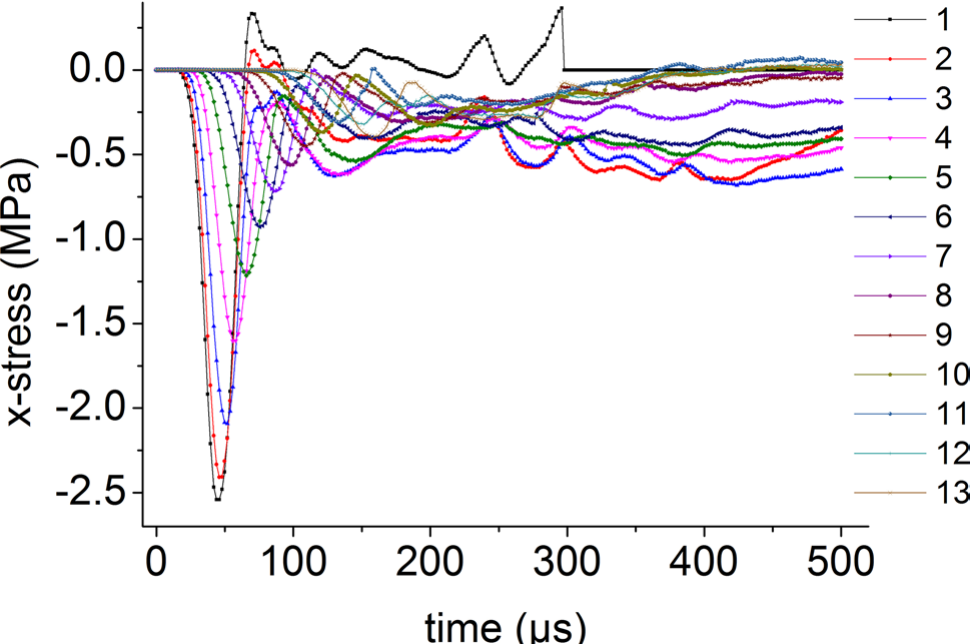
Radial stress and time relationship.

Thirteen element points are equidistantly selected from the center to the edge on the radial line of the coal mass. The curves for the axial stress and the radial stress when the impact bar speed is 10 m/s versus time are shown in Figs. 9 and 10, respectively. The axial stress peak and radial stress peak from the center to the edge on the radial line are continuously reduced.

**Figure 11:**
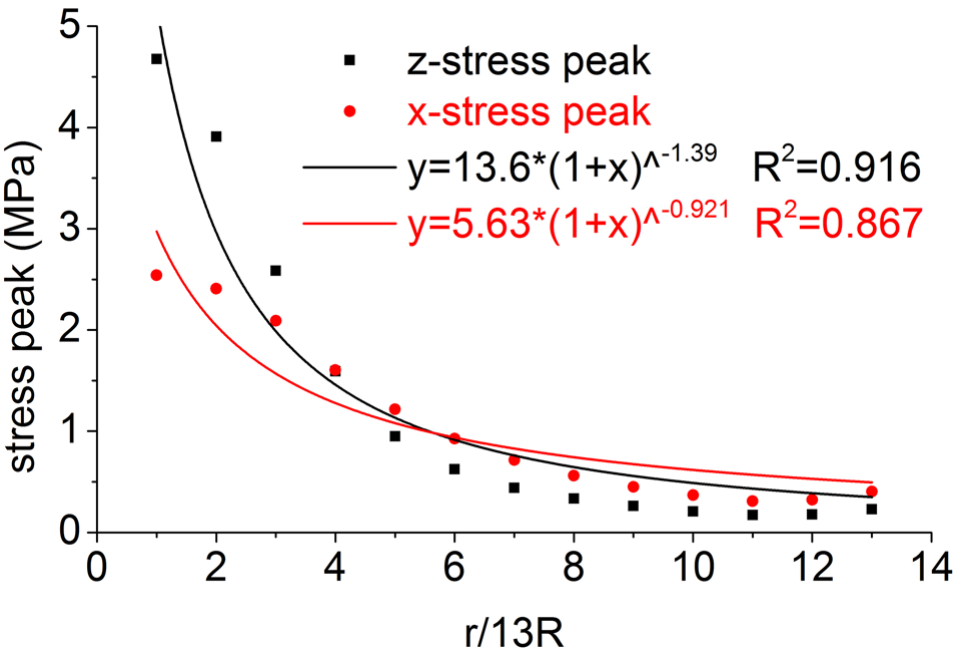
Distribution of the axial and radial stress peaks.

**Figure 12:**
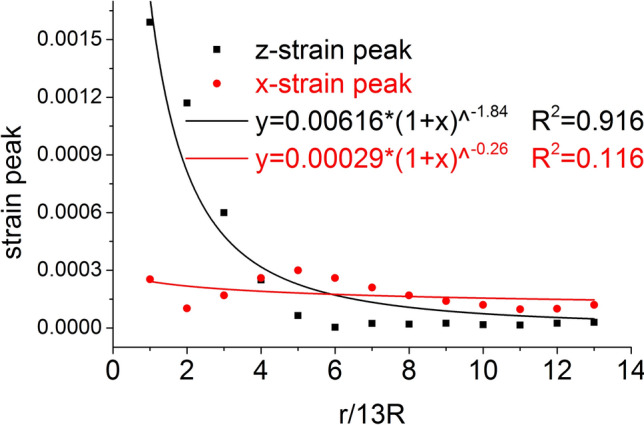
Distribution of the axial and radial strain peaks.

The distribution of the axial stress peak, radial stress peak, axial strain peak, and radial strain peak at thirteen element points on the radial line are shown in Figs. 11 and 12, respectively. The distribution of the axial stress peak, radial stress peak and axial strain peak at the radial line accords with the power function of the Pow2P2 function model, and the correlation coefficient squares (*R*^2^) are 0.916, 0.867 and 0.916 respectively. The radial strain distribution does not conform to the power function. At the same position, the axial stress peak and the radial stress peak are close, and the axial strain peak on the radial line is from far greater than the radial strain peak to close to.

### Axial and radial stress and strain distributions for 100 m/s

**Figure 13:**
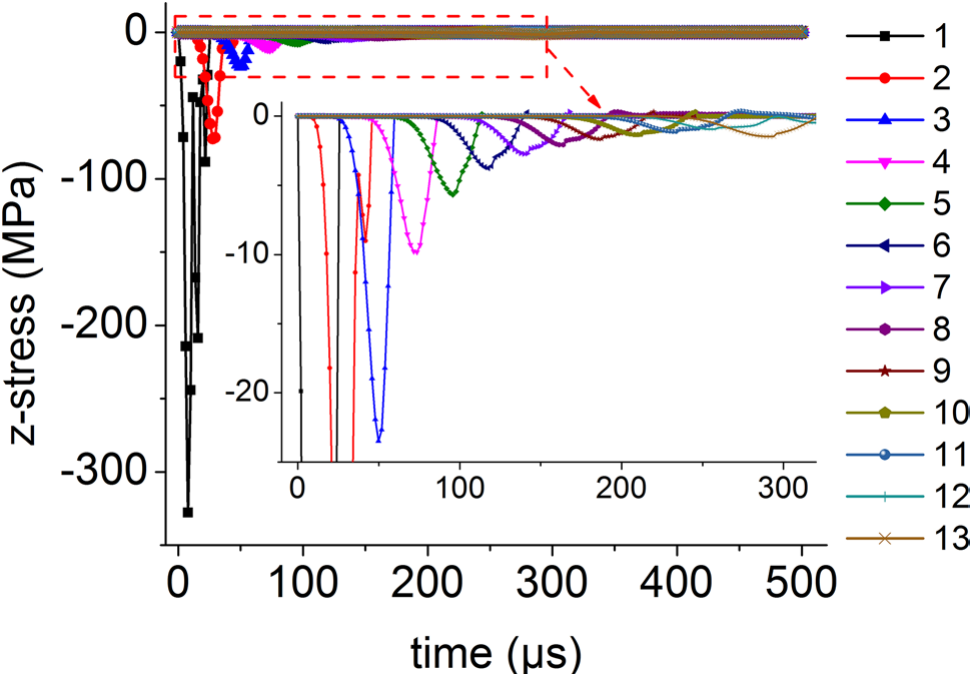
Axial stress and time relationship on the central axis.

On the central axis of the coal mass, thirteen element points are equidistantly selected from the top to the bottom. The curve for the axial stress when the impact bar speed is 100 m/s versus time is shown in Fig. 13. The axial stress peak on the central axis propagates from top to bottom, the axial stress peak decreases continuously and the axial stress behind the axial stress propagation direction is smaller than that at the front.

**Figure 14:**
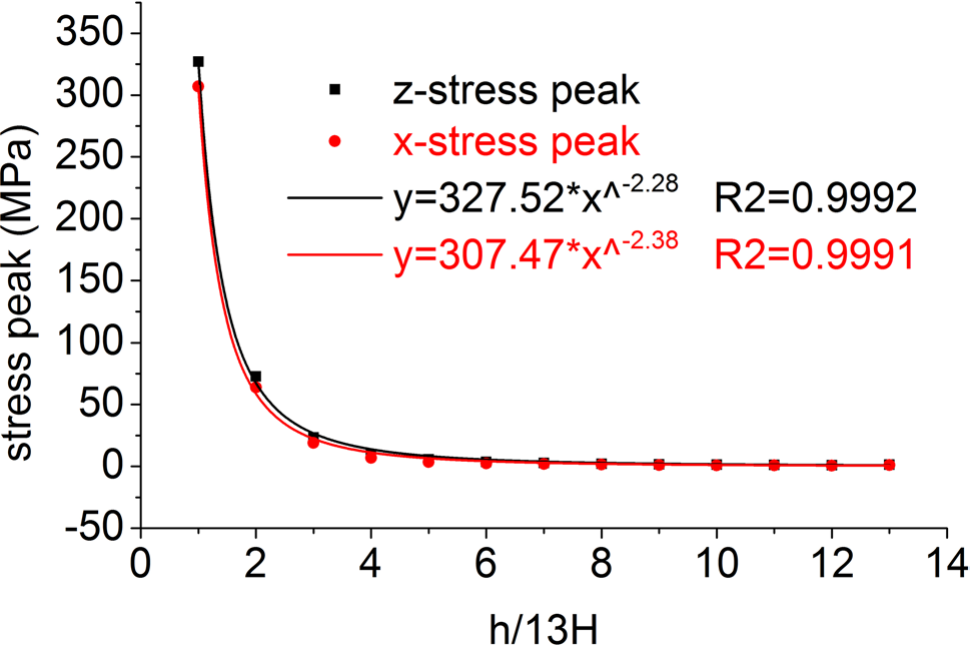
Distribution of the axial and radial stress peaks.

**Figure 15:**
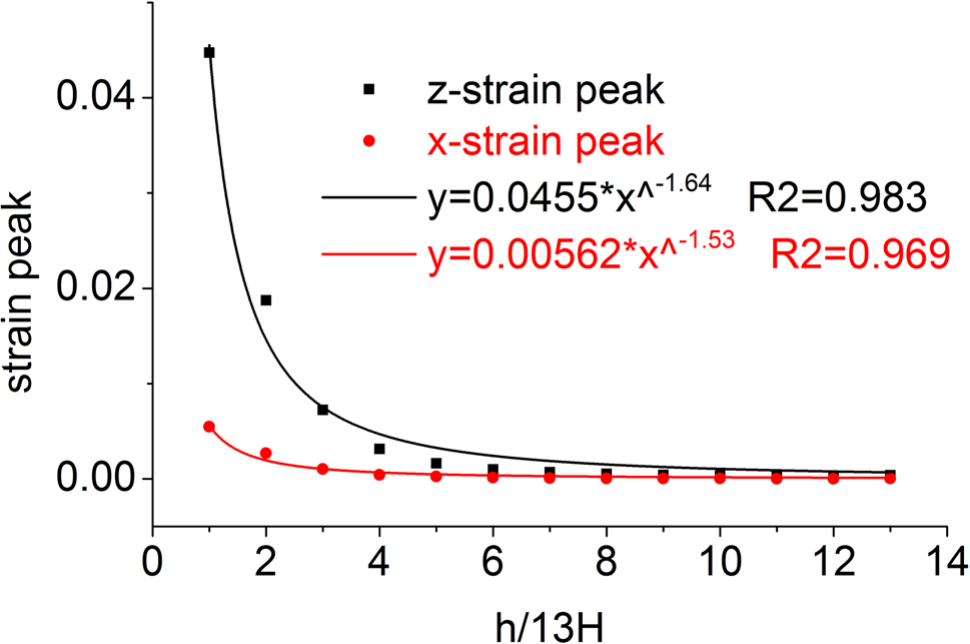
Distribution of the axial and radial strain peaks.

The distribution of the axial stress peak, radial stress peak, axial strain peak, and radial strain peak at thirteen element points on the central axis are shown in Figs. 14 and 15, respectively. The axial stress peak, radial stress peak, axial strain peak and radial strain peak distribution of the central axis are in accordance with the power function of the Allometric1 function model, and the correlation coefficient square (*R*^2^) is greater than 0.96. At the same position, the axial stress peak and the radial stress peak are close, and the axial strain peak on the central axis is from far greater than the radial strain peak to close to.

**Figure 16:**
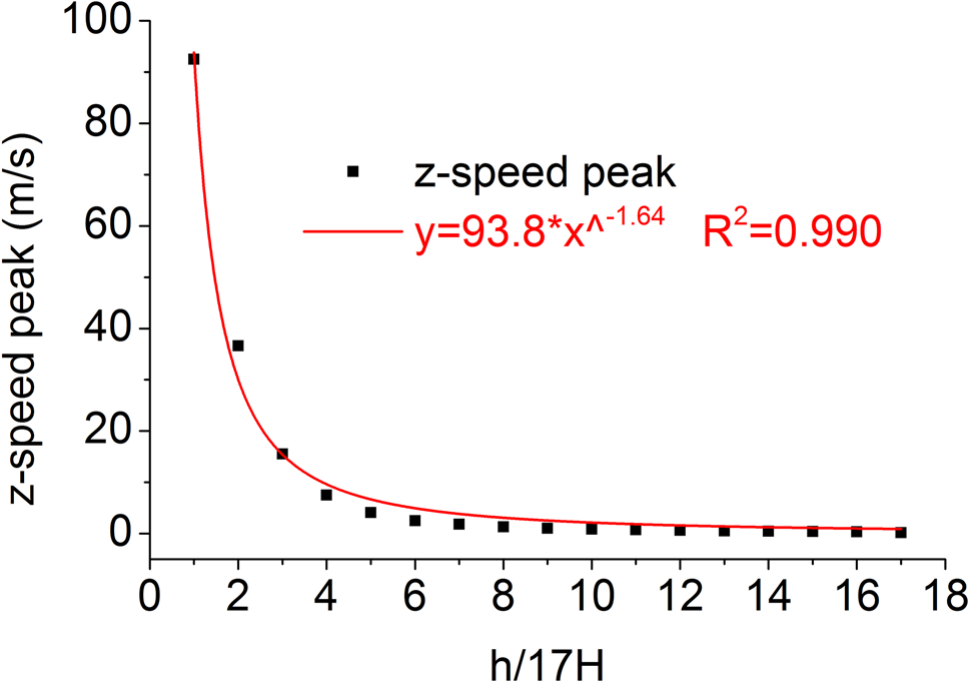
Distribution of the axial speed.

On the central axis of the coal mass, seventeen element points are equidistantly selected from the top to the bottom. The distribution of the axial speed peak at seventeen element points on the central axis when the impact bar speed is 100 m/s is shown in Fig. 16. The power exponent of the axial velocity peak is the same as the power exponent of the axial strain peak.

**Figure 17:**
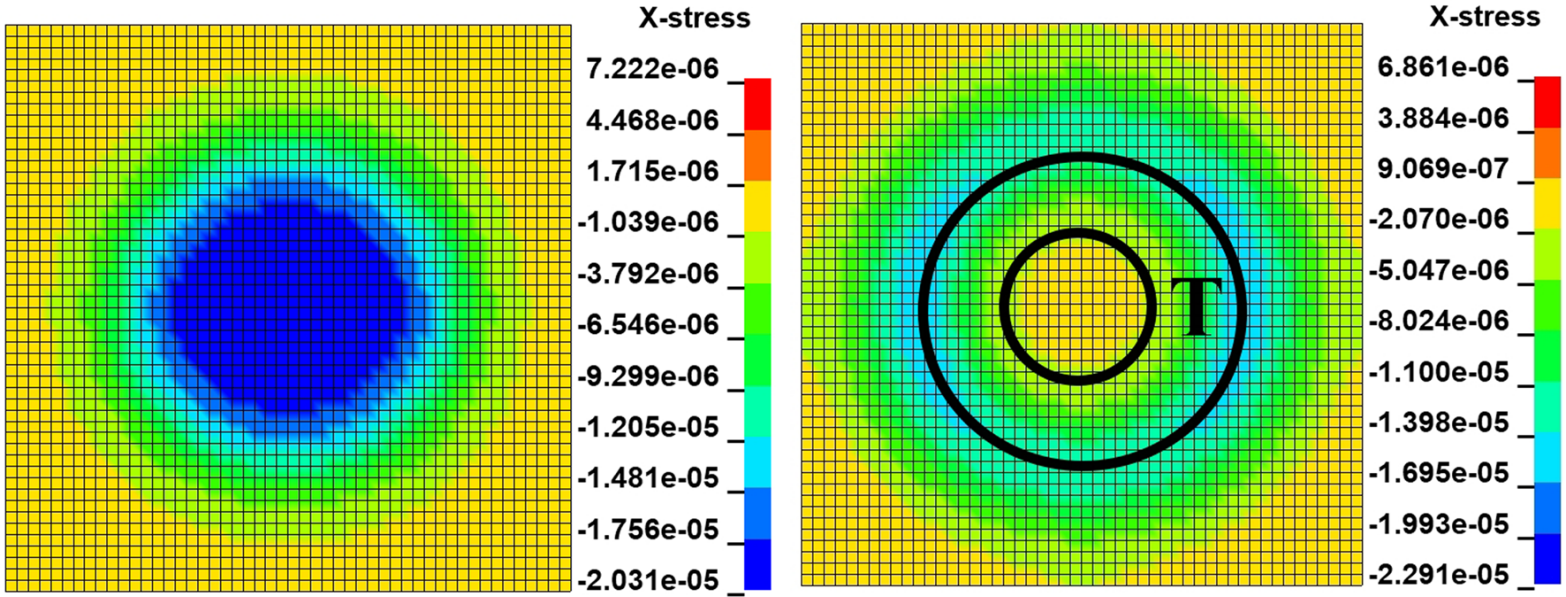
Dynamic distribution of the radial stress in the cross section at a velocity of 100 m/s.

The radial stress distribution on the cross section of the coal mass is shown in Fig. 17. The radial stress peak propagates from the center of the circle to the circumference. The change law of the radial stress is consistent with the change law at 10 m/s.

**Figure 18:**
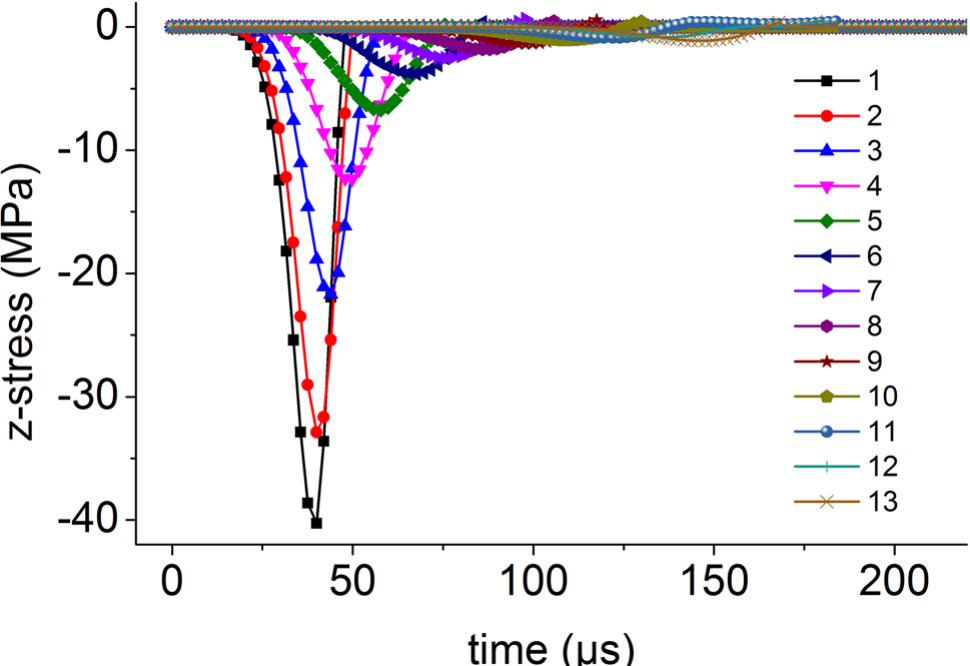
Axial stress and time relationship on a radial line.

Thirteen element points are equidistantly selected from the center to the edge on the radial line of the coal mass. The curve for the axial stress when the impact bar speed is 100 m/s versus time is shown in Fig. 18. The axial stress peak propagations from the center to the edge on the radial line.

**Figure 19:**
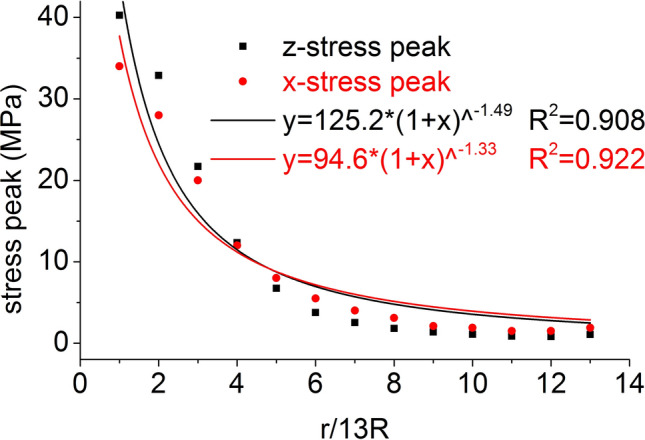
Distribution of the axial and radial stress peaks.

**Figure 20:**
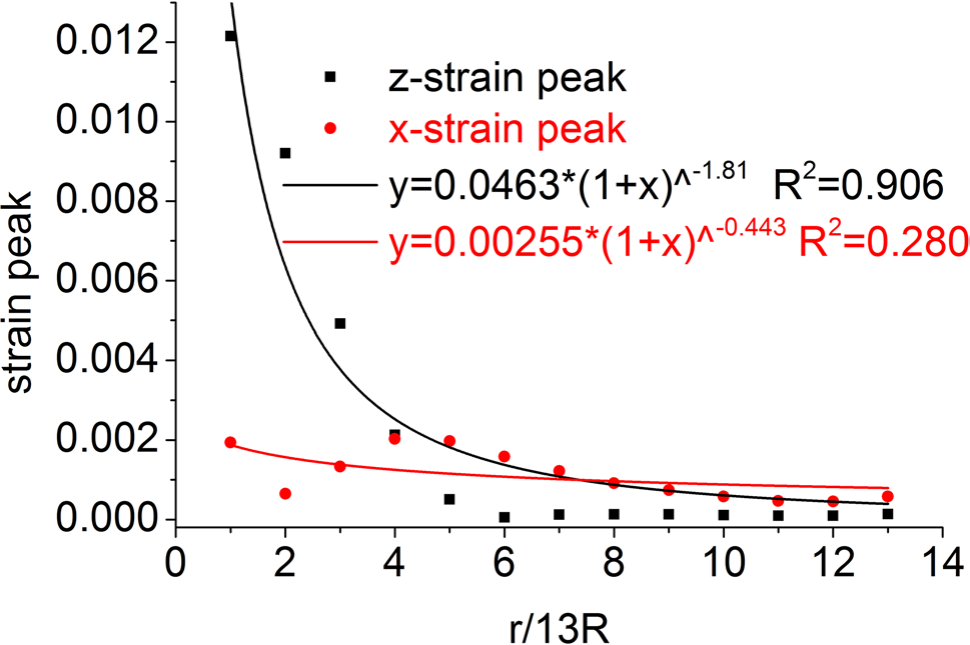
Distribution of the axial and radial strain peaks.

The distribution of the axial stress peak, radial stress peak, axial strain peak, and radial strain peak at thirteen element points on the radial line are shown in Figs. 19 and 20, respectively. The distribution of the axial stress peak, radial stress peak and axial strain peak at the radial line accords with the power function of the Pow2P2 function model, and the correlation coefficient square (*R*^2^) is greater than 0.9. The radial strain distribution does not conform to the power function. At the same position, the axial stress peak and the radial stress peak are close, and the axial strain peak on the radial line is from far greater than the radial strain peak to close to.

As the speed of the impact bar increases, the dynamic load becomes larger, the damage of the coal mass is more serious, and the laws of the central axis and the radial line are more obvious.

## Analysis of the stress, strain and energy

### Element point axial stress–strain

**Figure 21:**
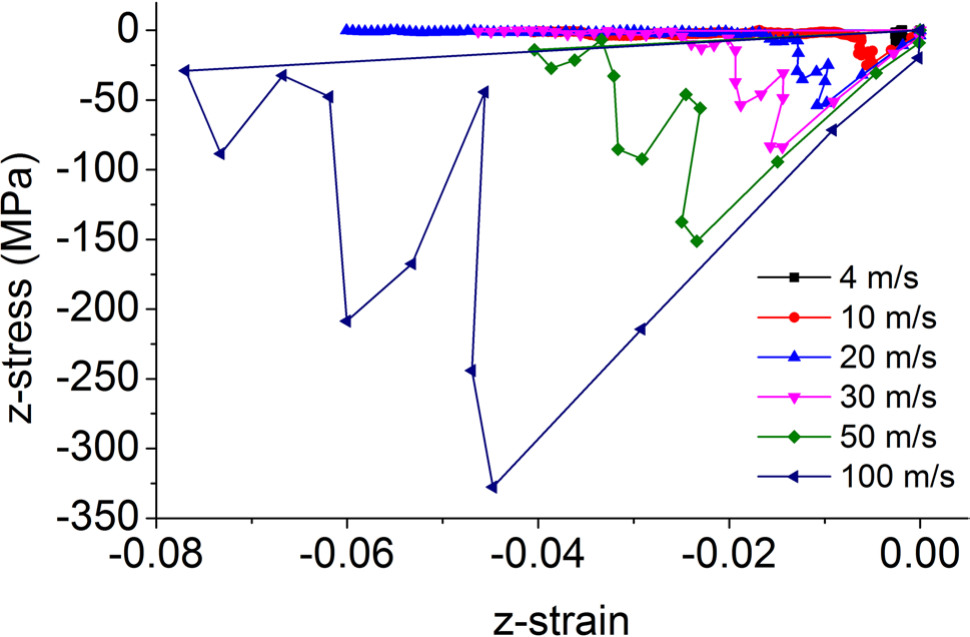
Element point axial stress–strain curve at the center of the force surface.

The element point axial stress–strain curves at the center of the force surface during dynamic damage of the coal rock mass are analyzed. The axial stress–strain curves of the element points are shown in Fig. 21 for various impact bar speeds.

The initial stage of the axial stress–strain curve at the center of the force surface conforms to the linear elastic variation law. After the stress reaches its peak, the coal mass undergoes damage, and the axial stress drops rapidly. At low speeds, the axial stress–strain change law is similar to the stress–strain curve of rock under static loads^64–66^. When the speed is large, the axial stress–strain curve is similar to the stress–strain curve of the coal under the dynamic load experiment. When the speed is large, after the axial stress reaches the maximum value, the axial stress decays rapidly, and the axial strain does not continuously increase. The final axial strain is zero due to element damage and failure.

**Figure 22:**
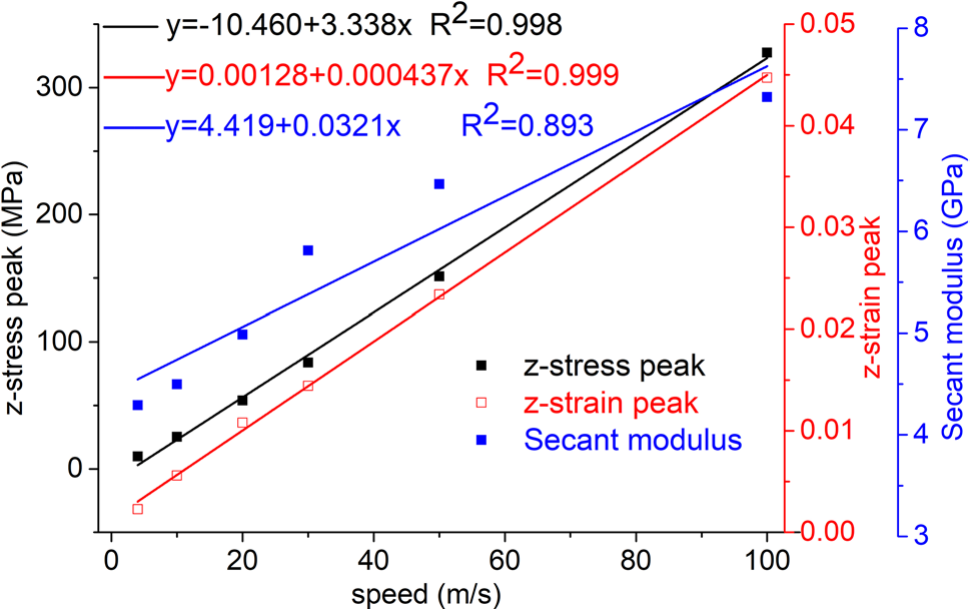
The fitting curve of the speed and axial stress peak, axial strain peak and secant modulus.

The secant modulus corresponding to the axial stress peak of the element point at the center of the force surface is analyzed. Taking the impact bar velocity as the independent variable, and the axial stress peak, axial strain peak and secant modulus of the element point at the center of the force-receiving surface as the dependent variables, the fitting curve is shown in Fig. 22.

The axial stress peak, axial strain peak and secant modulus increase with increasing velocity and have a linear relationship with the velocity, and the correlation coefficient squares (*R*^2^) are 0.998, 0.999 and 0.893, respectively. This indicates that the dynamic load can increase the axial stress peak, axial strain peak and secant modulus of the coal mass.

### Effective stress, effective strain and time

**Figure 23:**
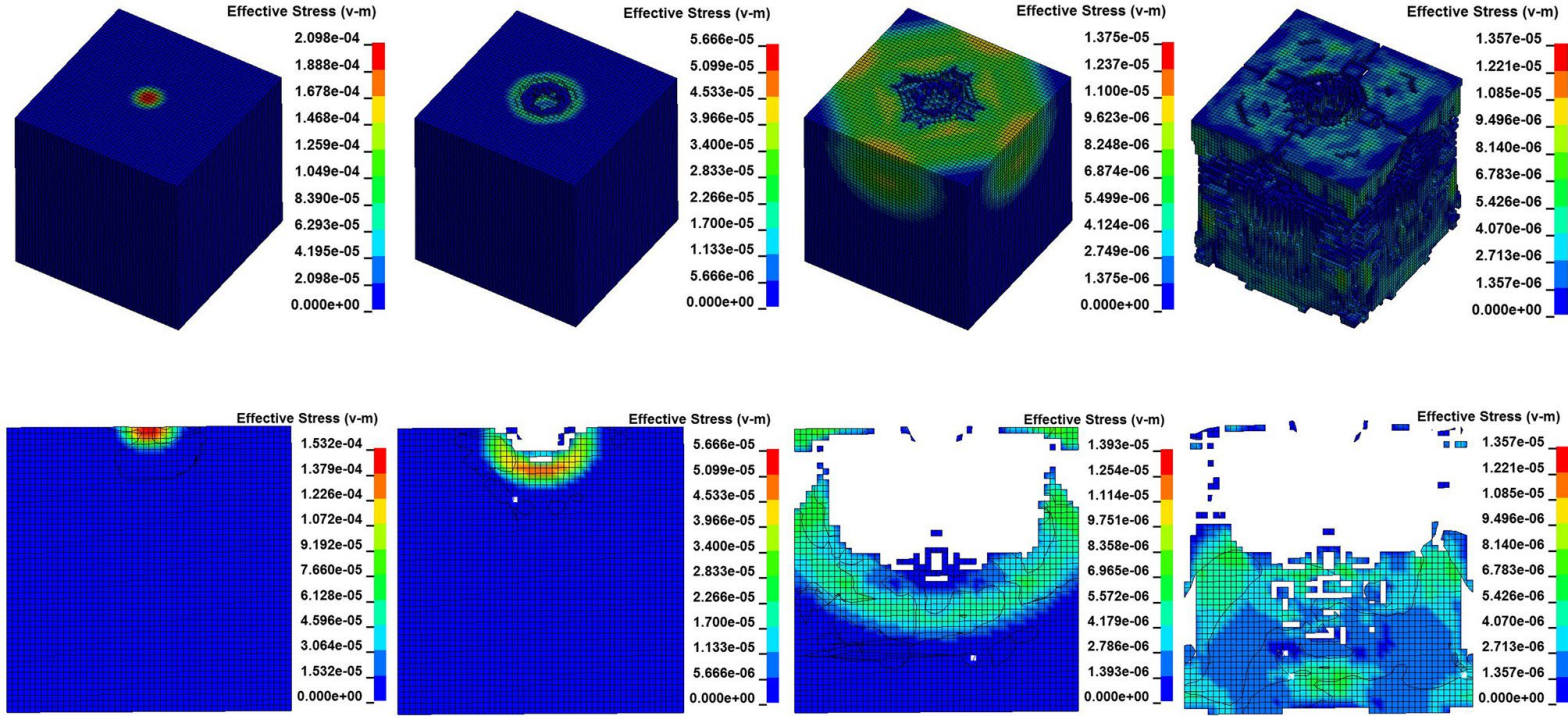
Dynamic distribution of the effective stress at an impact velocity of 100 m/s.

At each impact bar speed, the stress and morphological changes of the coal mass are similar (only when the velocity is 1 m/s does the coal mass not break), and the force and damage degree increase with increasing impact bar speed. The dynamic distribution of the effective stress at an impact velocity of 100 m/s is shown in Fig. 23.

The maximum effective stress of the coal mass is moved from the center of the contact surface sphere to the periphery, reaching the maximum stress at this location, which is called the maximum effective stress. Then, the coal mass fails and develops spherically downward. When the impact velocity is 100 m/s, the dynamic load is large, the coal mass is plastically deformed where the stress wave arrives, and the compressive stress is greater than the compressive stress of the coal mass, causing the coal rock mass to rupture.

**Figure 24:**
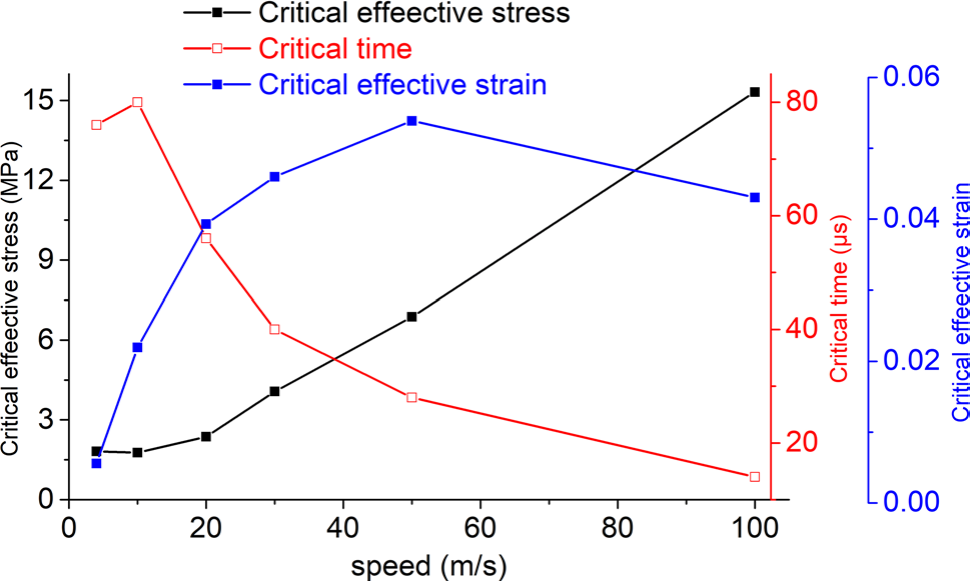
The relationship between the critical effective stress, critical effective strain, critical time and speed.

Under different impact bar velocities, the effective stress at the moment of failure of the coal mass is called the critical effective stress, the corresponding time is called the critical time and the corresponding effective strain is called the critical effective strain. Taking the impact bar velocity as the independent variable, and the critical effective stress, critical strain and critical time as the dependent variables, these relationships are shown in Fig. 24.

During dynamic loading, the critical effective stress of the coal mass increases with increasing impact speed, indicating that dynamic loading can change the effective stress of coal mass damage. The critical time decreases with increasing speed, indicating that the greater the velocity is, the faster the stress transfer. The critical effective strain increases with increasing impact velocity, and the increase gradually decreases. This indicates that fracture occurs after the strain of the coal-rock reaches a certain value, and the strain does not increase due to the failure of the unit.

### Energy analysis of the coal rock mass

**Figure 25:**
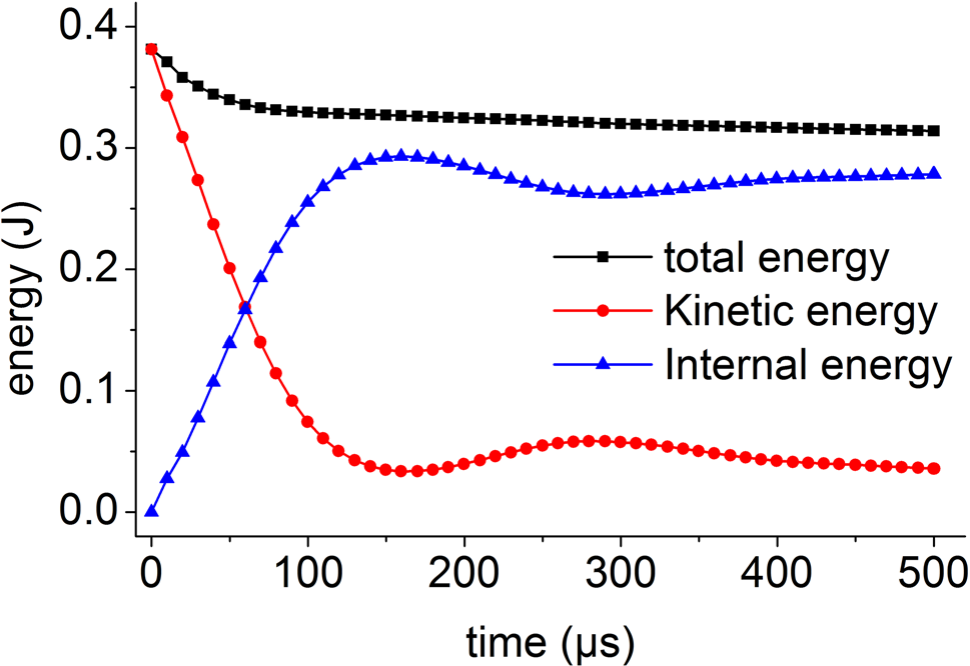
Energy time history at a velocity of 1 m/s.

The total energy of the coal mass is the sum of the internal energy and the kinetic energy of the coal mass. The energy time history of the coal mass at an impact bar velocity of 1 m/s is shown in Fig. 25. At this time, the coal mass does not break. The kinetic energy of the impact bar is transmitted to the coal mass by dynamic loading, the kinetic energy of the coal mass instantaneously reaches the maximum value, the kinetic energy of the coal mass is almost converted into internal energy, and the total energy decreases slightly with time.

**Figure 26:**
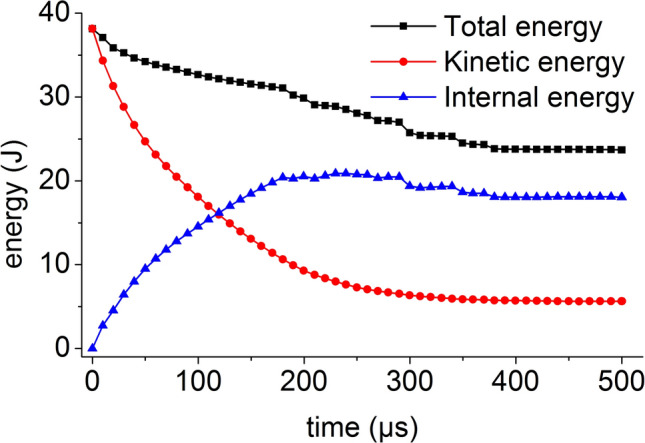
Energy time history at a velocity of 10 m/s.

When the impact bar speeds are 4 m/s and 10 m/s, the coal mass is slightly damaged, the kinetic energy of the coal mass is gradually reduced to the internal energy, and the conversion speed is also reduced. The total energy of the coal mass includes two forms of kinetic energy and internal energy. The total energy decreases slightly with time, and the energy time history is shown in Fig. 26 when the speed is 10 m/s.

**Figure 27:**
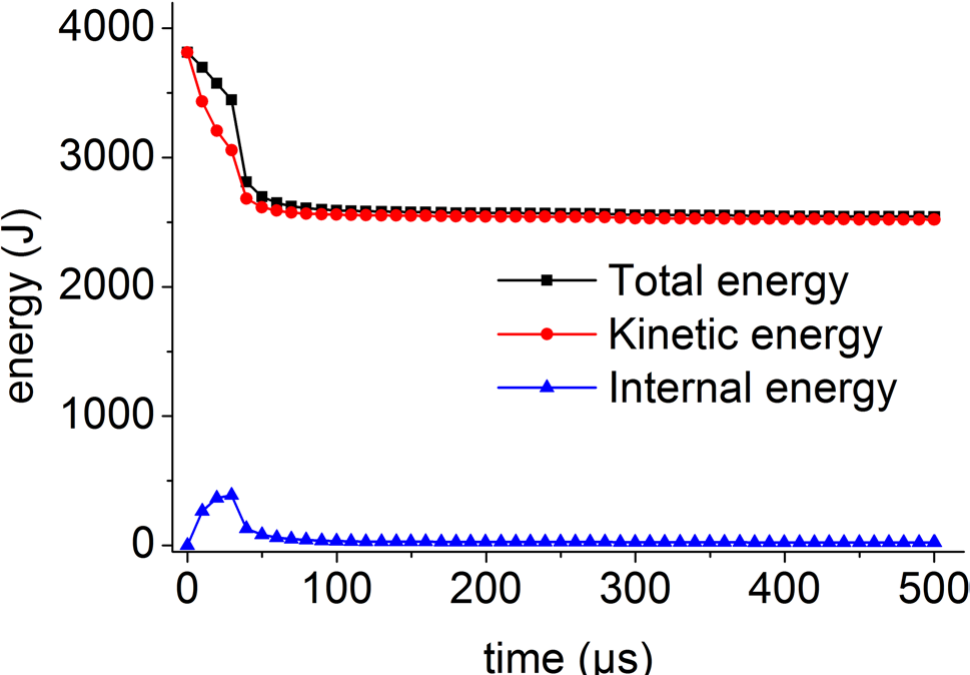
Energy time history at velocity of 100 m/s.

When the impact bar speeds are 20 m/s, 30 m/s, 50 m/s, and 100 m/s, the coal mass is seriously damaged, only a small part of the kinetic energy is converted into internal energy, and the total energy decreases slightly with time. The energy time history at a velocity of 100 m/s is shown in Fig. 27. When the speed is large, the coal mass undergoes severe deformation and destruction. After failure, most of the energy is in the form of kinetic energy, and the proportion of the internal energy is small.

**Figure 28:**
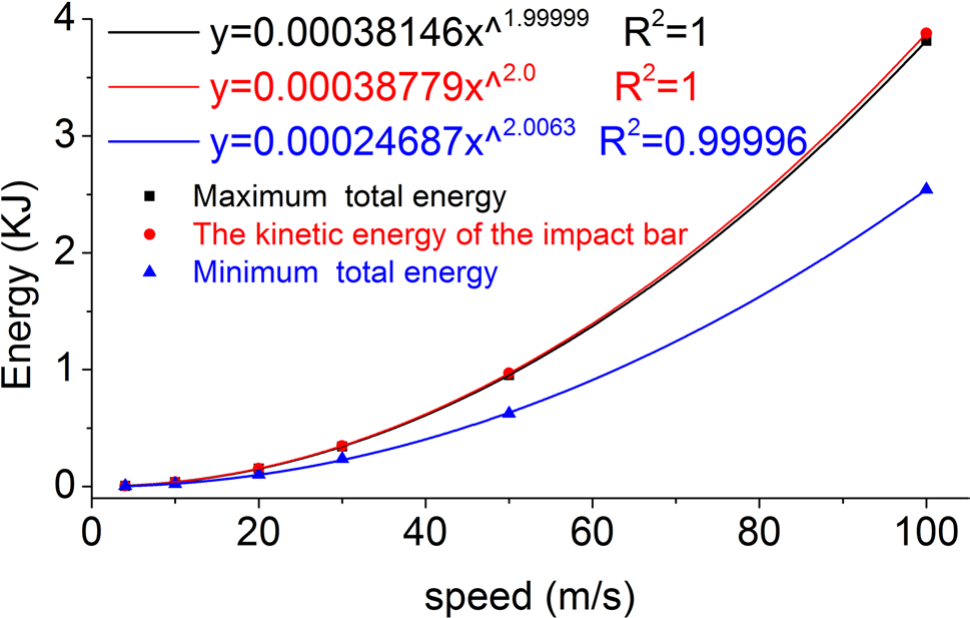
The fitting curve of speed and energy.

The maximum and minimum values of the total energy of the coal mass are called the maximum total energy and minimum total energy respectively. Equation (1) can be used to calculate the kinetic energy of the impact bar at each speed. Taking the impact bar velocity as the independent variable and the maximum total energy, minimum total and kinetic energy of the impact bar of the coal mass as the dependent variables, the fitting curve is shown in Fig. 28.

The maximum total energy, the minimum total energy, the kinetic energy of the impact bar and the velocity have a power function relationship that is in accordance with the Allometric1 function model, and the correlation coefficient squares (*R*^*2*^) are 1, 1 and 0.99996 respectively. Most of the kinetic energy of the impact bar is converted into the total energy of the coal rock mass. As the speed increases, the damage of the coal mass increases, the total energy loss also increases, the minimum total energy is roughly equal to the kinetic energy, the final kinetic energy also increases, the internal energy also increases, and the final internal energy is roughly equal to zero.

## Discussion

When the impact bar impacts the coal mass, the stress on the coal mass in contact with the impact bar is concentrated and plastic deformation occurs. Because: $\rho \nu {c}_{1}<\rho \nu {c}_{e}$, the stress behind the direction of propagation of the stress wave is smaller than that at the front, where tensile stress is generated and the coal mass is easily damaged.

Under the effect of different impact speeds, after the axial stress exceeds the peak at the apex, the axial stress generates tensile stress in the axial propagation direction, and after the radial stress exceeds the peak at the center point, the radial stress generates tensile stress in the radial propagation direction. The coal mass is prone to damage under tensile stress, and the tensile stress generated by numerical simulation is consistent with that of the theoretical analysis of the stress waves.

Under the effect of different impact speeds, the axial stress peak, radial stress peak and axial strain peak distribution of the central axis and radial line are in accordance with the power function model. At the same position, the axial stress peak and the radial stress peak are close, and the axial strain peak is from much larger than the radial strain peak to close to.

When the speed is large, the axial stress–strain curve is similar to the stress–strain curve of the coal under the dynamic load experiment^67,68^, and after the axial stress reaches a maximum value, the axial stress decays rapidly, and the axial strain does note continuously increase.

The initial total energy obtained by the coal mass in the numerical simulation is approximately equal to the kinetic energy of the impact bar calculated according to formula (1). As the speed increases, the damage of the coal mass increases, the total energy loss also increases, and the minimum total energy is roughly equal to the kinetic energy. The change law of the energy and kinetic energy in the coal mass is consistent with the damage law of the coal mass.

## Conclusion

At each impact bar speed, the stress and morphological changes of the coal mass are similar, and the force and damage degree increase with increasing impact bar speed.

1. Under the effect of different impact speeds, the axial stress peak, radial stress peak, and axial strain peak distribution of the central axis and the radial line are in accordance with the power function. The radial strain distribution does not conform to the power function. At the same position, the axial stress peak and the radial stress peak are close, and the axial strain peak is from much larger than the radial strain peak to close to.
2. Under the effect of different impact speeds, the axial stress generates tensile stress in the axial propagation direction, and the radial stress generates tensile stress in the radial propagation direction. The coal mass is prone to damage under tensile stress, and the tensile stress generated by the numerical simulation is consistent with that of the theoretical analysis of the stress waves.
3. When the speed is large, the axial stress–strain curve is similar to the stress–strain curve of the coal under the dynamic load experiment, and after the axial stress reaches a maximum value, the axial stress decays rapidly, and the axial strain does note continuously increase.
4. The axial stress peak, the axial strain peak and the secant modulus of the element points at the center of the stress surface have a linear relationship with the velocity. The dynamic load can increase the axial stress peak, axial strain peak and secant modulus of the coal mass.
5. The critical effective stress and the critical time have an approximately linear relationship with the impact bar velocity. When the strain of the coal mass reaches a certain value, cracking occurs, and the strain does not increase due to the failure of the unit.
6. The maximum total energy, minimum total energy and kinetic energy of the impact bar and the velocity have a power function relationship. Most of the kinetic energy of the impact bar is converted into the total energy of the coal mass. When the dynamic load is large, most of the energy is in the form of kinetic energy, and the total energy loss also increases.

## Data Availability

The primary data used to support the findings of this study are available from the corresponding author upon request.
